# Immune evasion strategies of bovine viral diarrhea virus

**DOI:** 10.3389/fcimb.2023.1282526

**Published:** 2023-10-13

**Authors:** Feng Pang, Qinqin Long, Min Wei

**Affiliations:** Department of Veterinary Medicine, College of Animal Science, Guizhou University, Guiyang, China

**Keywords:** bovine viral diarrhea virus (BVDV), immune evasion, innate immunity, adaptive immunity, therapies

## Abstract

Bovine viral diarrhea virus (BVDV) is a significant pathogen that causes great economic losses in the global livestock industry. During the long-term interactions between BVDV and its hosts, the virus has evolved multiple strategies to evade the host’s innate immunity and adaptive immunity, thereby promoting viral survival and replication. This review focuses on the most recent research on immune evasion strategies employed by BVDV, including evading type I IFN signaling pathway, evading host adaptive immunity, mediating NF-κB signaling pathway, mediating cell apoptosis and inducing autophagy. Unraveling BVDV’s immune evasion strategies will enhance our understanding of the pathogenesis of BVDV and contribute to the development of more effective therapies for the prevention, control and eradication of BVDV.

## 1 Introduction

Bovine viral diarrhea virus (BVDV) is a major pathogen responsible for bovine viral diarrhea (BVD), causing substantial economic losses in the global livestock industry (Lanyon et al., 2014; Oguejiofor et al., 2019; Al-Kubati et al., 2021). BVDV, border disease virus (BDV) and classical swine fever virus (CSFV) belong to *Pestivirus* genus of the *Flaviviridae* family which consists of 11 species named from *Pestivirus* A to *Pestivirus* K (Yesilbag et al., 2017; Gomez-Romero et al., 2021). BVDV exhibits great genetic diversity and is classified as three genotypes, BVDV-1 (*Pestivirus* A), BVDV-2 (*Pestivirus* B) and BVDV-3 (*Pestivirus* H or HoBi-like pestivirus). BVDV-1 can be classified into at least 21 sub-genotypes (BVDV1a-1u) while BVDV-2 and BVDV-3 can be classified into three (BVDV 2a-2c) and four (a-d) sub-genotypes, respectively (de Oliveira et al., 2020; Nugroho et al., 2022). According to the lytic activity of the virus in cell culture, BVDV strains of each genotype are divided into two distinct biotypes, cytopathic (cp) and non-cytopathic (ncp) (Peterhans et al., 2003). Most natural infection and persistent fetal infection are caused by ncp isolates, whereas cp isolates are uncommon and primarily associated with fatal BVD outbreaks referred to as mucosal disease(MD) (Brackenbury et al., 2003).

**Figure 1:**
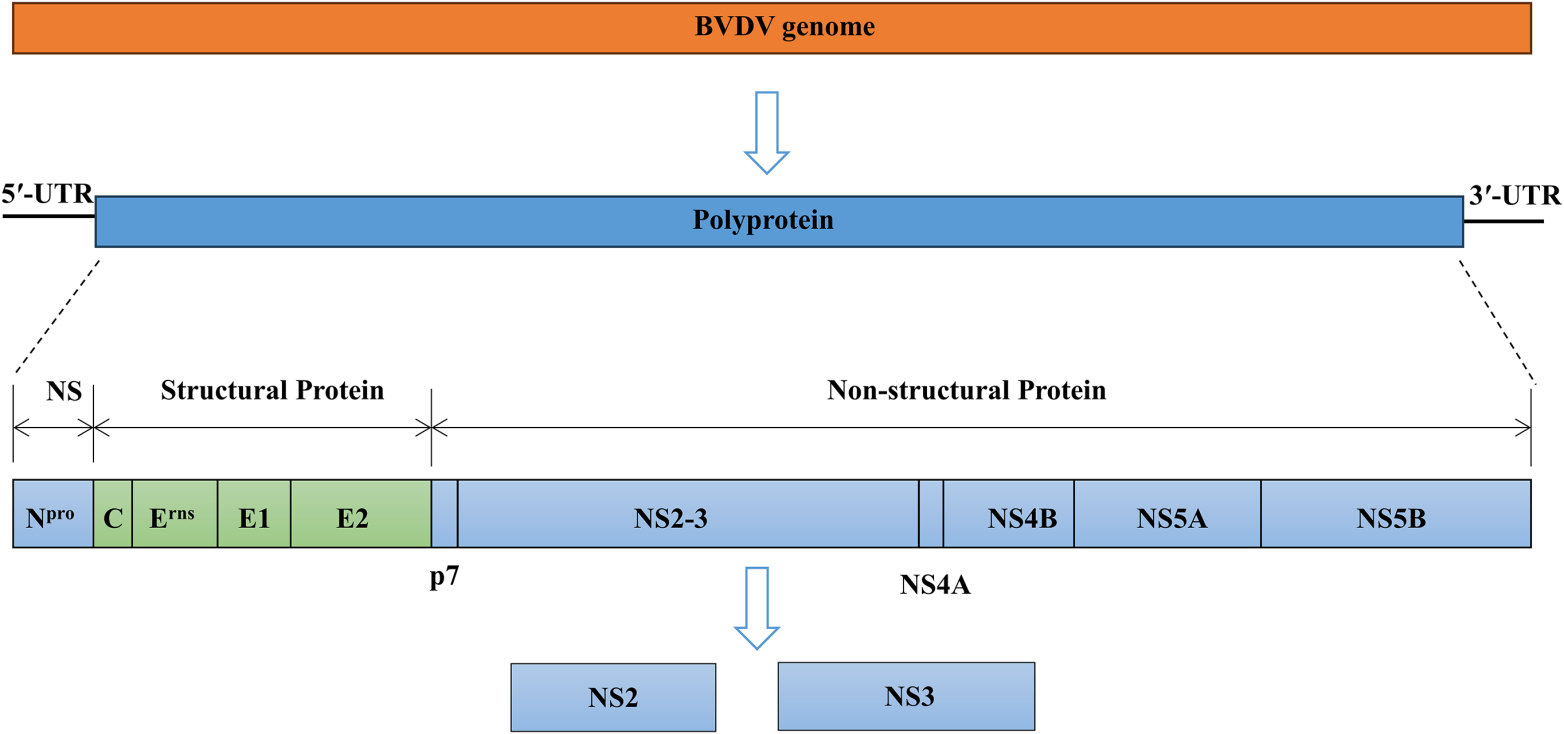
Overview of the genome organization of BVDV virion. BVDV encodes a single large polyprotein that is post-translationally cleaved into four structural proteins (C, E^rns^, E1, and E2) and eight non-structural (NS) proteins (N^pro^, p7, NS2, NS3, NS4A, NS4B, NS5A, and NS5B).

BVDV is a small, enveloped, single-stranded, positive-sense RNA virus. The BVDV particles are spherical in shape with a diameter of approximately 50 nm and consist of a core region of genomic RNA coated with the capsid or core protein (C). The core is surrounded by an outer lipid bilayer envelope in which three envelope proteins E^rns^, E1, and E2 are inserted into the membrane (Neill, 2013; Chi et al., 2022). The size of the BVDV genome ranges from 12.3 kilobases to 12.5 kilobases and consists of a single large open reading frame (ORF) flanked by short 5′- and 3′-untranslated regions (UTRs) (Chi et al., 2022; Hong et al., 2023). The ORF encodes a single large polyprotein that is post-translationally cleaved into four structural proteins (C, E^rns^, E1, and E2) and eight non-structural proteins (N^pro^, p7, NS2, NS3, NS4A, NS4B, NS5A, and NS5B) (**Figure 1**) (Mirosław and Polak, 2019; King et al., 2021). The cp BVDV is characterized by the constructive expression of NS3, while ncp BVDV strains produce a fusion of NS2 and NS3 proteins named NS2-3 (Darweesh et al., 2015). BVDV infection causes variable symptoms including growth retardation, hemorrhaging, respiratory and gastrointestinal disorders, reproductive failures, persistent infection, and lethal mucosal disease (MD) (Mirosław et al., 2022; Zhu et al., 2022).

Throughout the co-evolutionary process between BVDV and its hosts, the virus has devised multiple tactics to evade host immune defense. This article summarizes the latest research on how BVDV evades host immune response, aimed to determine potential new targets and develop novel therapies for the prevention and control of BVDV infection.

## 2 BVDV evades host innate immunity

### 2.1 Innate immune sensing of BVDV

The innate immune system is the host’s first line of defense against invading pathogens. It responds quickly to infection by recognizing common pathogen characteristics, in contrast to the adaptive immune system, which takes days to generate adequate activated antigen-specific lymphocytes. Upon infection, the host′s innate immune response is initiated by various pattern recognition receptors (PRRs). PRRs recognize and bind to pathogen-associated molecular patterns (PAMPs), triggering a signaling cascade that ultimately leads to the production of type I interferon and other inflammatory cytokines (Zhu and Zheng, 2020; Roetman et al., 2022; Chen et al., 2023). The major PRRs for sensing RNA viruses are Toll-like Receptors (TLRs), RIG-I-like receptors (RLRs), C-type lectin receptors (CLRs) and NOD-like receptors (NLRs)(Chow et al., 2018; Wang et al., 2021b).

#### 2.1.1 TLR-mediated recognition of BVDV

TLRs are important innate receptors expressed in a wide variety of mammalian immune and non-immune cells. TLR3, TLR7, TLR8 and TLR9 are expressed in intracellular vesicles such as the endosome and the endoplasmic reticulum (ER) while other TLRs are expressed on the cell surface. TLR3 recognizes double-stranded RNA (dsRNA) while TLR7 and TLR8 recognize single-stranded RNA (ssRNA) produced by viruses during viral infection (Bayraktar et al., 2019; de Marcken et al., 2019). When monocytes were infected with ncp BVDV, the mRNA levels of TLR3, type I IFN and IL-12 were significantly increased at 1 h post infection, whereas the TLR7 mRNA was significantly upregulated in monocytes infected with both biotypes at 24 h post infection (Lee et al., 2008). Moreover, the mRNA levels of TLR3, TLR7 and CD86 were significantly increased in spleen and tracheobronchial lymph node on day 5 post inoculation with high virulence ncp BVDV, but not the low virulence strains (Palomares et al., 2014).

#### 2.1.2 RLR-mediated recognition of BVDV

RLRs include three members, retinoic acid-inducible gene I (RIG-I), melanoma differentiation-associated protein 5 (MDA5) and laboratory of genetics and physiology 2 (LGP2). RIG-I recognizes 5’-di/triphosphate-containing, short, panhandle double-stranded RNA and activates downstream signaling pathway in an ATP-dependent manner. RIG-I has a preference for short viral dsRNA, whereas MDA-5 binds selectively to long dsRNA. Both RIG-I and MDA5 interact with the adapter molecule mitochondrial antiviral signaling protein (MAVS), activating RLR signaling pathway, inducing the production of proinflammatory cytokines and type I IFN. The RLRs play a critical role in sensing of RNA virus infection to initiate and modulate antiviral immunity (Jensen and Thomsen, 2012; Onomoto et al., 2021; Zhang et al., 2022). Based on lncRNA sequencing results and lncRNA-mRNA co-expression network, researchers found that differentially expressed lncRNAs in cp BVDV-infected MDBK cells were mainly enriched in apoptosis, NOD-like receptor (NLR) and RIG-I-like receptor (RLR) signaling pathways (Gao et al., 2021; Gao et al., 2022).

### 2.2 Host innate immune response to BVDV infection

Interferons (IFNs) are generally classified into three types: type I, type II, and type III IFNs (Hoffmann et al., 2015). They show distinct expression patterns and are involved in innate and adaptive immunity in a variety of ways. Type I and type III IFNs are primary antiviral IFNs. Type I IFNs are composed of IFN-α (13 subtypes in humans and 14 subtypes in mice), IFN-β, IFN-ϵ, IFN-κ, IFN-ω (humans) and IFN-ξ (mice). In mammals, the type I IFN response is essential for innate antiviral responses. Binding of IFN-α/β to its receptor (IFNAR) activates downstream Janus kinase (JAK)-signal transducers and activators (STAT) signaling pathway, ultimately leading to the induction of large numbers of IFN-stimulated genes (ISGs) to trigger a global antiviral state (Lazear et al., 2019; Song et al., 2022). Type II IFNs include only one member, IFN-γ, which is secreted by activated T cells, natural killer (NK) cells, NKT cells, and dendritic cells (Rojas et al., 2021). Generally, type II IFNs act as a bridge between the innate and adaptive immune responses. Type III IFNs (IFN-λ1, IFN-λ2, IFN-λ3 and IFN-λ4) are genetically different from type I IFNs, but they activate antiviral pathways similar to type I IFNs. The type III IFN receptor is mainly restricted to epithelial cells, while the type I IFN receptor is present on all nucleated cell types (Mesev et al., 2019).

It has been reported that acute infection with ncp BVDV resulted in a significant increase in bovine serum IFN-γ and IFN-α levels. Specifically, in the peripheral blood mononuclear cells (PBMCs) of BVDV-infected cows, classical IFN-activated signaling pathways, such as JAK-STAT were activated and ISGs were significantly induced (Van Wyk et al., 2016). Moreover, cp BVDV infection induced IFN-β production in MDBK cells by activating interferon regulatory factor 1 (IRF1), IRF7, and NF-κB signaling pathways in a way similar to poly I:C stimulation (Maldonado et al., 2020). Infection with cp BVDV was found to induce the activation of dsRNA-dependent protein kinase (PKR) that phosphorylated alpha subunit of the translational initiation factor (eIF2α), leading to translation inhibition while ncp BVDV did not induce PKR activity and eIF2α phosphorylation, aiming to establish a persistent infection (Gil et al., 2006b). However, the impact of BVDV on the JAK-STAT signaling pathway remained largely unknown, which was required for further studies.

### 2.3 BVDV evades type I IFN signaling pathway

For successful infection, most pestiviruses have evolved two proteins, the non-structural protein N^pro^ and the envelope glycoprotein E^rns^ to prevent the activation of innate immunity (De Martin and Schweizer, 2022). E^rns^ has been reported to inhibit dsRNA-mediated signaling pathways, depending on its dsRNA binding properties and RNase activity (Iqbal et al., 2004). E^rns^ had the ability to inhibit type I IFN production induced by viral dsRNA or ssRNA, due to its RNase activity to degrade viral RNA(Mätzener et al., 2009; Zürcher et al., 2014). N^Pro^ was sufficient to suppress IFN-β induction by targeting IRF3 for polyubiquitination and proteasomal degradation, without relying on its autoprotease activity (Hilton et al., 2006). Others showed that cp BVDV infection did not induce detectable levels of IFN-α/β and that the N-terminal domain of N^pro^ protein was required for type I IFN antagonism, independent of its catalytic endoproteinase activity (Gil et al., 2006a). Furthermore, the ncp BVDV N^pro^ protein was determined to interact with the cellular S100A9 protein and reduce its activity in infected cells, leading to reduced type I IFN production, thereby promoting viral replication (Darweesh et al., 2018).

The cp BVDV NS4B protein significantly inhibited RLR-mediated IFN-β promoter activity as well as MDA5 mRNA levels (Shan et al., 2021). Furthermore, NS4B protein was shown to inhibit the MDA5-mediated signal transduction by acting as an IFN-β antagonist through direct interaction with two N-terminal caspase-recruitment domains (CARDs) of MDA5. In addition, BVDV infection induced higher mRNA and protein levels of DNA damage-inducible transcript 3 (DDIT3), which inhibited type I IFN production by regulating MAVS degradation during viral infection in MDBK cells and in mice (Wang et al., 2021a).

**Figure 2:**
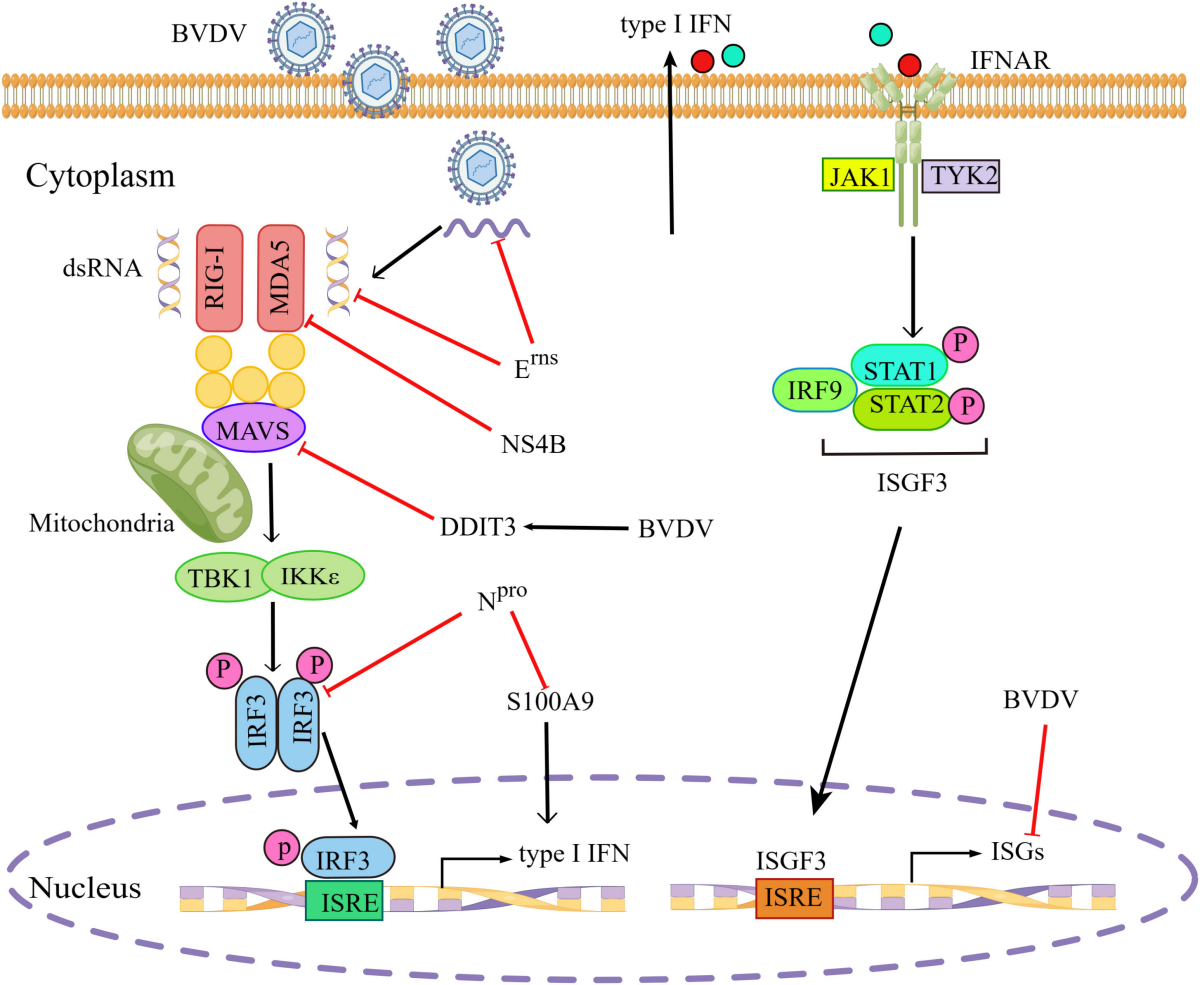
BVDV evades type I IFN signaling pathway. BVDV has evolved various strategies to evade type I IFN signaling pathway by inhibiting multiple steps in the signal transduction. BVDV, Bovine viral diarrhea virus; RIG-I, retinoic acid-inducible gene I; MDA5, melanoma-differentiation-associated gene 5; MAVS, mitochondrial antiviral-signaling protein; TBK1, TANK-binding kinase 1; IKKϵ, inhibitor of IκB kinase ϵ; IRF3/9, interferon regulatory transcription factor 3/9; IFNAR, interferon-α/β receptor; JAK1, janus kinase 1; TYK2, tyrosine kinases 2; STAT1/2, signal transducer and activation of transcription 1/2; ISGs, interferon-stimulated genes; P, phosphate; ISRE, interferon-stimulated response element; ISGF3, interferon-stimulated gene factor 3; DDIT3, DNA damage-inducible transcript 3; S100A9, S100 calcium binding protein A9. Red blunt end arrows indicated inhibition of the signaling pathway by BVDV or BVDV proteins. Black solid arrows indicated pathway connection. The figure was drawn by Figdraw.

High-throughput sequencing, such as RNA-seq, is a powerful tool to study viral pathogenesis and virus-host interaction. A RNA-Seq-based transcriptome analysis of MDBK cells infected with BVDV suggested that ncp BVDV infection significantly inhibited the expression of several antiviral ISGs, such as ISG15, Mx1, and OSA1Y, which were involved in the inhibition of host′s innate immunity at the early stage during BVDV infection (Liu et al., 2019). Another similar study showed that cp BVDV infection significantly upregulated two negative NF-κB regulators, IER3 and TNFAIP3, thus possibly blocking this signaling pathway (Villalba et al., 2016). The evasion of type I IFN signaling pathway by BVDV is illustrated in **Figure 2**.

## 3 BVDV evades host adaptive immunity

### 3.1 BVDV impairs antigen presentation and evades cellular immunity

The innate immune system is the first line of defense against various pathogens. Adaptive immune cells then mount antigen-specific responses and establish long-term memory immunity. Previous evidence indicated that cp BVDV infection resulted in a significant reduction in the expression of the Fc receptor (FcR) and the complement receptor (C3R) in alveolar macrophages, which are required for phagocytic activity, thereby decreasing antigen uptake and presentation (Welsh et al., 1995). The effect of BVDV infection on antigen presentation was dependent on viral strains and/or the maturity of antigen presenting cells (APCs). Both monocytes and dendritic cells (DCs) were susceptible to two biotypes BVDV infection, but monocytes were lysed by cp BVDV while DCs were not (Glew et al., 2003; Chase, 2013). Furthermore, monocytes infected with ncp BVDV failed to stimulate CD4^+^ T cell responses while DCs remained unaffected. When monocytes were differentiated into monocyte-derived dendritic cells (Mo-DCs), Mo-DCs lost the ability to produce infectious virions, but were able to produce viral RNA, as well as viral NS5A and E2 proteins, likely due to the interruption of virus packaging or release process (Rajput et al., 2014). In addition, ncp BVDV downregulated the expression of MHC-I, MHC-II, and CD86 on Mo-DC surface, which may account for the immunosuppression and persistent infection caused by ncp BVDV (Rajput et al., 2014). Proteomic profiling revealed that cp BVDV infection significantly decreased the expression of 9 MHC-I proteins and 6 MHC-II proteins in bovine monocytes compared to mock-infected controls, thereby inhibiting antigen presentation to immunocompetent T cells (Lee et al., 2009). Bovine CD4^+^ T cells played a critical role in controlling BVDV infection. Evidence suggested that depletion of CD4^+^ T cells in calves exposed to ncp BVDV resulted in an increase in blood viral load and prolonged duration of viremia, whereas depletion of either CD8^+^ T cells or γδ^+^ T cells had no observable effect(Howard et al., 1992). Both CD4^+^ and CD8^+^ T cell proliferation was elicited from virus-infected stimulatory cells whereas only CD4^+^ T cells responded to non-infectious BVDV antigens, including E^rns^, E2, NS2-3, capsid and/or N^pro^ proteins (Collen and Morrison, 2000).

### 3.2 BVDV evades humoral immunity

The glycoprotein E^rns^ of BVDV, a highly glycosylated envelope protein, induced significant antibody production in animals. However, the neutralizing activity of antibodies targeting E^rns^ was limited (Donis, 1995). E2 was the largest glycoprotein and antigenic target for the induction of neutralizing antibodies (Liang et al., 2005; Li et al., 2013; Cheng et al., 2023). After natural infection or inoculation with a modified live vaccine, the B cell response, as measured by serum antibody levels, was predominantly directed against viral E2 and NS2/3 proteins, with lesser responses directed against E^rns^ and E1 proteins. Vaccination with killed vaccines led to the production of serum antibodies directed primarily against the E2 protein (Ridpath, 2013; Griebel, 2015; Al-Kubati et al., 2021).

When calves were infected with cp BVDV, total IgG and IgG1 concentrations were significantly decreased at 7 days post infection (DPI) and then recovered by 21 DPI (Rajput et al., 2020). Ncp BVDV infection did not affect total IgG concentration at 7, 14 and 21 DPI until an obvious rise occurred from 21 to 35 DPI. For both biotypes, the IgG1 concentration and the IgG1:IgG2 ratio were significantly reduced from 0 DPI to 7 DPI. Calves infected with ncp BVDV had significantly higher IgG1 levels at all time points and higher IgG2 concentrations at 7 and 35 DPI compared to cp TGAC strain. Ncp BVDV induced significantly higher levels of serum neutralizing antibodies at 14, 21 and 35 DPI than cp BVDV, suggesting that ncp BVDV evoked a stronger immune response than cp BVDV.

## 4 BVDV mediates NF-κB signaling pathway

NF-κB is a key transcriptional complex that regulates the expression of genes involved in inflammation and the immune response (Zhang et al., 2017; Reus et al., 2022). A previous study showed that cp BVDV infection induced the production of various inflammatory cytokines and antiviral molecules in MDBK cells. Interestingly, all BVDV activities were inhibited by pharmacological NF-κB pathway inhibitors. Infection with cp BVDV promoted NF-κB activation and p65 translocation to the nucleus within minutes of virus entry into cells (Fredericksen et al., 2015). More recently, scientists identified that cp BVDV infection resulted in the activation the NF-κB signaling pathway and enhanced the expression of NLRP3 inflammasome components and inflammatory cytokine pro-IL-1β post BVDV infection (Fan et al., 2022). As a result, pro-caspase-1 and pro-IL-1β were cleaved into their active forms, caspase-1 and IL-1β. Further experimental results indicated that viral NS5A and E^rns^ proteins were involved in the BVDV-induced inflammatory response by activating the NF-κB signaling pathway.

## 5 BVDV mediates cell apoptosis

Ncp BVDV strains did not cause cell death, but were capable of establishing persistent infection, whereas cp BVDV strains induced cytopathic effects (CPE) via apoptosis in bovine cells. Cp BVDV infection led to the activation of the intrinsic apoptotic pathway, which was characterized by the translocation of cytochrome c from mitochondria to the cytosol, Apaf-1 overexpression, significantly increased caspase-9 activity and the formation of apoptosome (Grummer et al., 2002). It was found that the mRNA levels of apoptosis-associated genes such as Mx1, iNOS, TNF-α and OAS1 were upregulated following cp BVDV infection (Yamane et al., 2006). RNAi silencing of both PKR and OAS-1 resulted in almost complete inhibition of apoptosis induced by cp BVDV infection without affecting viral titers. In conclusion, dsRNA was a major inducer of apoptosis in cp BVDV-infected cells whereas ncp BVDV produced minimal levels of dsRNA without induction of apoptosis to establish a persistent infection. Furthermore, overexpression of cp BVDV NS3 protein alone or NS3Δ50 (deleting N- terminal 50 amino acids) truncated protein was able to induce apoptosis *in vitro* that correlated with initiator caspase-8 and caspase-9 activation (St-Louis et al., 2005). Cp BVDV NS3/4A induced an intrinsic apoptotic pathway characterized by increased activity of caspase-9 and caspase-3 in MDBK cells dependent on NS3 protease activity (Gamlen et al., 2010). Apart from an intrinsic pathway, cp BVDV can also induce apoptosis through an extrinsic pathway by upregulating TNF-α and iNOS mRNA levels and inducing the expression of both TNFR-1 and TNFR-2, which was required to convert an extrinsic factor signal into an intracellular death signal(Yamane et al., 2005).

Moreover, cp BVDV induced endoplasmic reticulum (ER) stress-induced apoptosis in MDBK cells (Jordan et al., 2002). Cp BVDV activated the ER transmembrane kinase PERK and caused phosphorylation of the translation initiation factor eIF2α, which induced CHOP/GADD153 expression and consequently led to the downregulation of anti-apoptotic Bcl-2 protein and a decrease in intracellular GSH levels. Finally, cp BVDV infection induced the expression of caspase-12 and initiated apoptosis. In a recent study, cp BVDV was demonstrated to trigger ER stress-related apoptosis in bovine placental trophoblast cells (BTCs), thereby promoting the spread of BVDV (Yuan et al., 2023). Inhibition of ER stress-induced apoptosis blocked BVDV replication and significantly reduced BVDV virulence. To sum up, cp BVDV induced apoptosis *via* the intrinsic pathway, the extrinsic pathway and the ER stress-mediated apoptosis signaling pathway.

## 6 BVDV induces autophagy in various cell types

Autophagy, a catabolic process of great significance, plays a critical role in the maintenance of cellular homeostasis (Klionsky et al., 2021). There are three types of autophagy: macro-, micro-, and chaperone-mediated autophagy (CMA). Macro-autophagy, referred to as “autophagy”, is the major pathway (Khandia et al., 2019). A previous study has demonstrated that infection with cp BVDV induced autophagy and significantly upregulated the mRNA and protein levels of autophagy-related genes, Beclin1 and ATG14 in MDBK cells at 12 h post infection (Fu et al., 2014a). Nevertheless, the knockdown of Beclin1 and ATG14 did not impact cp BVDV infection-related autophagy. The viral envelope proteins E^rns^ and E2 were responsible for the induction of autophagy by increasing the formation of autophagosomes, the percentage of GFP-LC3 puncta-positive cells and the protein levels of Beclin1 and ATG14 (Fu et al., 2014b). Both biotypes had the ability to induce autophagy in MDBK and primary bovine turbinate (Bt) cells at similar levels and used autophagy to replicate themselves (Rajput et al., 2017). Similarly, ncp BVDV infection induced autophagy in MDBK cells by significantly increasing the conversion of LC3-I to LC3-II, upregulating the autophagy-related proteins ATG5 and Beclin 1 and degrading p62/SQSTM1, thereby promoting viral replication(Shin et al., 2023). The BVDV non-structural protein NS4B alone was able to induce autophagosomes in bovine kidney LB9.k cells, representing a novel and significant function of NS4B in BVDV replication (Suda et al., 2019). Two biotypes of BVDV utilized different strategies to regulate the unfolded protein response (UPR) mechanisms and ER stress-mediated autophagy for their own benefit (Wang et al., 2023). In an unconventional manner, the cp BVDV downregulated the expression level of the ER chaperone GRP78, thereby promoting self-replication through the UPR, while the ncp BVDV retained activating transcription factor 4 (ATF4) in the cytoplasm to provide an advantage for its persistent infection.

## 7 Conclusions and perspectives

BVDV has evolved multiple immune evasion strategies to establish persistent infection and facilitate its transmission. Understanding these mechanisms is critical for the development of effective vaccines and control measures against BVDV. Further research is needed to clarify the molecular mechanisms responsible for BVDV-induced immunosuppression and discover novel targets for antiviral intervention. In addition, it is crucial to develop advanced vaccines that offer comprehensive defense against various strains of BVDV in order to minimize the influence of this economically important pathogen on the livestock industry.

## Author contributions

FP: Conceptualization, Funding acquisition, Visualization, Writing – original draft, Writing – review & editing. QL: Conceptualization, Writing – review & editing. MW: Writing – review & editing.
